# Evaluating the implementation of a national disclosure policy for large-scale adverse events in an integrated health care system: identification of gaps and successes

**DOI:** 10.1186/s12913-016-1903-7

**Published:** 2016-11-11

**Authors:** Elizabeth M. Maguire, Barbara G. Bokhour, Todd H. Wagner, Steven M. Asch, Allen L. Gifford, Thomas H. Gallagher, Janet M. Durfee, Richard A. Martinello, A. Rani Elwy

**Affiliations:** 1 Center for Healthcare Organization and Implementation Research, Edith Nourse Rogers Memorial Veterans Hospital, Bedford, MA USA; 2 Department of Health Law, Policy and Management, Boston University School of Public Health, Boston, MA USA; 3 Health Economics Resource Center, VA Palo Alto Healthcare System, Menlo Park, CA USA; 4 Center for Innovation to Implementation, VA Palo Alto Healthcare System, Menlo Park, CA USA; 5 Stanford University School of Medicine, Palo Alto, CA USA; 6 University of Washington School of Medicine, Seattle, WA USA; 7 Patient Care Services, Veterans Health Administration, Department of Veterans Affairs, Washington, DC USA; 8 Yale School of Medicine, New Haven, CT USA; 9 Center for Healthcare Organization and Implementation Research, VA Boston Healthcare System, 150 S. Huntington Ave, Jamaica Plain, MA USA; 10 Center for Healthcare Organization and Implementation Research, 200 Springs Road (Mailstop152), Bedford, 01730 MA USA

**Keywords:** Adverse events, Veterans, Qualitative, Communication

## Abstract

**Background:**

Many healthcare organizations have developed disclosure policies for large-scale adverse events, including the Veterans Health Administration (VA). This study evaluated VA’s national large-scale disclosure policy and identifies gaps and successes in its implementation.

**Methods:**

Semi-structured qualitative interviews were conducted with leaders, hospital employees, and patients at nine sites to elicit their perceptions of recent large-scale adverse events notifications and the national disclosure policy. Data were coded using the constructs of the Consolidated Framework for Implementation Research (CFIR).

**Results:**

We conducted 97 interviews. Insights included how to handle the communication of large-scale disclosures through multiple levels of a large healthcare organization and manage ongoing communications about the event with employees. Of the 5 CFIR constructs and 26 sub-constructs assessed, seven were prominent in interviews. Leaders and employees specifically mentioned key problem areas involving 1) networks and communications during disclosure, 2) organizational culture, 3) engagement of external change agents during disclosure, and 4) a need for reflecting on and evaluating the policy implementation and disclosure itself. Patients shared 5) preferences for personal outreach by phone in place of the current use of certified letters. All interviewees discussed 6) issues with execution and 7) costs of the disclosure.

**Conclusions:**

CFIR analysis reveals key problem areas that need to be addresses during disclosure, including: timely communication patterns throughout the organization, establishing a supportive culture prior to implementation, using patient-approved, effective communications strategies during disclosures; providing follow-up support for employees and patients, and sharing lessons learned.

**Electronic supplementary material:**

The online version of this article (doi:10.1186/s12913-016-1903-7) contains supplementary material, which is available to authorized users.

## Background

Implementing a national policy for disclosing large-scale adverse events to patients is a complex process. Large-scale adverse events are not unusual [1]. This type of event occurs when a significant number of patients are affected by a breakdown in care in a healthcare setting [2]. A common example is when medical equipment, such as endoscopes, is reprocessed incorrectly between patient use. Patients are potentially exposed to infectious diseases such as HIV and viral hepatitis, though the risk is often very low or uncertain [3]. Healthcare organizations struggle with whether and how to notify patients when there is no known disease transmission [4]. Disclosures of these adverse events seek to give patients an accurate description of the risk, while maintaining their trust, by communicating both what is known and unknown about the event [5]. Patients respond best to full disclosure of adverse events [6]. Some healthcare organizations like the Veterans Health Administration (VA) have mandates to notify patients of these events in an effort to be transparent and maintain trust in addition to ensuring that each patient involved in the adverse event is able to take any necessary action for their health and well-being.

Many healthcare organizations have developed disclosure policies, including the VA, to guide these disclosure communications [8, 9]. However, much of the adverse event disclosure research has focused on individual clinical events [1, 7, 10] while large-scale adverse event disclosure communications have not been well shared [9].

Patients expect to be notified quickly about adverse events [7]. However, many healthcare organizations are unprepared to respond quickly and effectively to large-scale events. Delays in notification may occur due to the time it takes to 1) recognize the adverse event, 2) evaluate the scope of the event and the identities of the individuals exposed and 3) for leadership to decide to disclose after discussion with subject matter experts and other stakeholders. The development of an organizational disclosure policy is the first step in expediting notification. Some adverse events involve thousands of exposed patients and coordination of timely review, communication, and testing can be a daunting task requiring substantial efforts [11, 12]. It is important to evaluate both how the disclosure policy is communicated to leaders and employees who will conduct the disclosure communications, and to also look at the impact of these communications, both positive and negative, on patients, families and employees to evaluate the broad impact of such policies.

We report a retrospective, observational study involving interviews with stakeholders from past large-scale events to identify key problematic areas that may impact implementation of this national policy. We sought to identify barriers and facilitators to this implementation, specifically among patients, employees, leadership, and other stakeholders. We also sought to understand whether elements of the disclosure communication and organizational culture had an effect on policy implementation.

## Methods

### Facility selection

In the United States healthcare system where most insurance is private, VA is the largest integrated healthcare system. The VA, run by the government, operates approximately 150 hospitals with over 1000 outpatient sites of care. Each hospital is led by a Facility Director. At the time this study was conducted, VHA was organized into 21 regions across the United States and each region was led by a Network Director. All regions, hospitals and clinics are expected to follow national policy set by the Central Office located in Washington DC. For this study we reviewed all large-scale adverse events across the system and identified nine VA medical facilities with these types of events since 2009 (Table 1). The events were identified by the VA’s Office of Public Health, who assisted in selecting nine events that were timely and representative of the types of events occurring in the healthcare organization. In addition, representatives from the Centers for Disease Control (CDC) were part of our study Expert Panel and lent their expertise to the event selection process. The types of events varied from improper cleaning of endoscopes to inadequate infection control processes from a single provider to improper reuse of insulin pens by nursing staff. The number of potentially affected patients in the exposure group for each event ranged from 168 to 6805.

**Table 1:** Description of Nine Large-Scale Adverse Events for Analysis

Facility	Number of Patients Included in Disclosure	Interviews with Leaders	Interviews with Employees	Interviews with Patients and Family Members
Site 1	1104	2	–	–
Site 2	6805	2	–	–
Site 3	2531	3	12	6
Site 4	1812	2	10	8
Site 5	535	2	5	–
Site 6	982	2	1	–
Site 7	716	2	5	5
Site 8	286	2	5	8
Site 9	168	3	–	–
Congressional staff members	4 interviews			
VA Central Office Leaders	8 interviews			

### Qualitative interviews

**Fig. 1:**
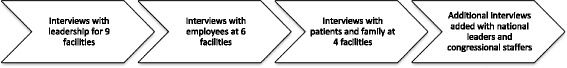
Description of the staged interview process

Qualitative, semi-structured interviews began with Network Directors and other regional leaders, followed by Facility Directors from each of the nine sites (see Fig. 1). These interviews took place by phone and lasted 30 min (See Additional file 1 for interview guides). Some Facility Directors expressed concern about interviewing employees and patients at their sites. These adverse events were upsetting for many staff and patients involved and some facilities felt that interviews would be disruptive to those who were still in the process of recovering from the event. As a result, we did not interview employees and patients at all nine sites. Employee interviews were conducted at six sites, followed by interviews of Veterans/patients and their family members at four sites. The four sites that participated in all levels of interviews represented a variety of adverse events impacting both small and large patient populations. Participants received a screening survey by email (employees) or mail (patients) and indicated their willingness to participate. Employee and patient interviews took place primarily in person, with some phone interviews as needed. These interviews were 45–60 minutes in length and were recorded after consent was obtained.

While conducting these interviews, we found that there were many aspects of communication and decision-making related to VA’s disclosure policy where we needed additional information to fully understand the implementation of the disclosure policy. Therefore, we conducted additional phone and in-person interviews with VA Central Office leadership in Washington, DC to address these gaps. These, in turn, led us to adding interviews with staff members representing elected officials who were part of the U.S. Congressional Veterans Affairs committees, who are responsible for providing oversight to the Department of Veterans Affairs, a role analogous to a board of directors or trustees for other private or public organizations.

Our interview guide was tailored for each role: leader, employee, patient, family member, or Congressional staff member. Interviews began with how the participant learned about the large-scale adverse event and then moved to questions about positives and negatives of the communication, the implementation process, and the lasting effects.

### Conceptual framework

We used the Consolidated Framework for Implementation Research (CFIR) as a guide for identifying constructs important to large-scale adverse event disclosure [12]. This approach has been applied to other VA policy and program implementations, as well as in non-VA settings [12, 13]. The CFIR covers a full spectrum of dissemination and implementation elements and can be used in diverse applications [14, 15]. The CFIR describes five major domains, involving 26 subconstructs within these domains, which have the potential for both serving as barriers or facilitators to any implementation: 1) characteristics of the intervention, 2) the outer setting of an organization, 3) the inner setting, 4) characteristics of individuals involved, and 5) the process of implementation [14, 15]. These constructs were derived from multi-disciplinary domains, including psychology and organizational change [14].

### Data analysis

Our analysis and reporting follow accepted guidelines for qualitative research [16]. Following the interviews, the team took field notes and the recorded interviews were transcribed. Interview transcripts and notes were first coded using a grounded thematic approach [17] and the analysis of this data has been previously published [18]. The coding team, consisting of three researchers (EM, ARE, BB), identified many implementation themes not previously reported in our prior publication. We then recoded all interviews using the CFIR in a complementary analysis that showed new findings for healthcare leadership. The coding team met biweekly to discuss application of the CFIR coding framework to the interview data to ensure consistency. The CFIR allows us to apply only the relevant subconstructs to our interview data [12]. In each of the CFIR five domains there are key constructs which made up our coding frame. The team met regularly to resolve any discrepancies in coding. From this review, we identified subconstructs from the CFIR that were most prominent and were related to positive or negative large-scale adverse event disclosure policy implementation experiences.

## Results

Seven CFIR subconstructs within three domains were most prominent in interviews, either for positive or negative influence on implementation. In the inner setting domain: networks & communication and culture; in the intervention characteristics domain: design of notification & packaging, execution and costs; and in the process domain: engaging external stakeholders and reflecting & evaluating (Table 2).

**Table 2:** Key CFIR Constructs and Illustrative Quotes

Domain	Construct	Interview Quote (s)	Key Lessons Learned
Inner Setting	Networks & Communication	“It took weeks to wait on approval for the letters. It ended up impacting the perception of hiding information.” –Facility Director	Existing networks and communications patterns, positive or negative, will impact the ability to organize and disclosure in a timely way.
	Culture	“I think as far as what, clinically how we moved forward, I think a lot of, um, what I’ve done next in my team is t-to remind people to s-, ya know, speak up if you see something that doesn’t seem quite right.” –Facility Director	Establish supportive culture focusing on open communication and importance of transparency prior to implementation.
Intervention Characteristics	Design of Notification & Packaging	“I liked getting the phone call because it kept me from being shocked, like shocked about getting AIDS or something. They reassured me, they said just come down and we’ll check it out.” –Patient	Patients prefer phone call first.
	Execution	“I think we shoulda been told (the testing results) a lot sooner that, that’s my only still, it’s not a complaint or a blame game.” –Patient	Minimize layers of approval and time delays to move quickly.
Process	Engaging External Stakeholders	“I would have involved stakeholders earlier. Give them a heads up. You might not have all the answers but you can say to them, this is what we know so far and we’ll follow through. Give them lots of updates.” –Facility Leadership	Involve stakeholders as early as possible and maintain communication throughout.
	Reflecting & Evaluating	“We’ve just bashed our head against the wall so many times with this and it’s just silly that, you know, we, that there isn’t a central clearing house of information - a set of, you know, recommendations everyone can follow.” -Facility employee	Hold formal evaluations and sharing of lessons learned within the organization following a large-scale disclosure.

### Inner setting: networks & communication

Interviews with leaders and employees at all levels included discussion of the impact, both positive and negative, of networks and communications on the implementation of the disclosure policy and resulting patient notifications. One example was how employees found out about the adverse event and patient communication process. Small groups of frontline employees were included early on to assist with the notification/disclosure planning process. For most, this was their first experience with the VA’s large-scale adverse event disclosure policy and the resulting patient communication about this type of event. These employees at times felt they were in a silo and only received limited information. Employees who were not part of these work groups found out about the event much later. For some, they were made aware of the event by media coverage instead of communication from hospital leaders. Employees relied on informal communications channels when they did not receive formal communications from leaders: *“And there were a lot of different stories. The communication was not clear. I think everyone was uh, basically running for the hills so to speak uh, and the people that did know about it wouldn’t really communicate about it. The people that didn’t know about it relied on rumors.”-Facility employee.*

There were issues with communication identified that related to the many levels of involvement required for a large healthcare organization like the VA. When the policy is executed, there are many levels of review before approval is given which can result in delays and issues with communication. After facilities received official word that a large-scale adverse event would need to be communicated to patients, there were additional levels of approval needed for the vetting of the communications plan and messages and execution of patient disclosure*: “We had standing calls every day- with the (regional network) at 4:30 and with (national leaders) after that. We spent a lot of time waiting for approval.” -Facility leadership.*

Interviewees at all levels of the organization discussed their frustrations with communication among the many offices. Some suggested changes to the concurrence process, like having the full group participate in an initial meeting to make decisions, in order to shorten the review and approval time. Facilities also asked for more independence in carrying out the implementation of the patient communication and follow-up responses to stakeholders.

Many discussed the need for the development of formal communications with employees to share information and clear up any misunderstandings: *“Someone always knows dirt and can always leak information so you can’t control that. So you need to act quickly. Get the message to each employee quickly so they know what to say when they get approached. Arm them with the facts.”* -Facility leadership. In thinking about the future, a few staff identified the communications techniques of a new hospital Director as important. This leader sends weekly emails to all staff with current information about hospital events and information. Staff felt these open communications would serve the facility well if they faced another event in the future because they trusted that this leader would communicate with them.

### Inner setting: culture

Culture includes the norms and values of the healthcare organization [14]. Many interviews included discussion about disclosure policy given the low risk of the events studied. For many, the open disclosure to patients matched their desired culture of a “patient-first” organization. They expressed pride in the intervention: *“I was honored to be part of this important work and get a chance to do right by Veterans.” -Facility employee.*

One facility experienced a lot of leadership transition just before and during the implementation of the disclosure. As a result, the culture did not feel as certain with new leaders in place and led to communication and trust issues during disclosure. Others talked about how strong leadership is important before an adverse event occurs, and that leaders should be present and visible in the facility: *“And emails are sometimes overrated and you know what we could do, what, you know what often is, you know we have this um, this front line the, e- executive office say, ‘Let’s get out and make friends.’ They need to know who medical center director is. And don’t just show up when it’s a bad time.” -Facility employee.*

Others discussed the potential impact the disclosure policy and the experience of disclosure communications during a large-scale adverse event could have on the cultural values of improvement and future reporting of errors. Due to negative experiences, especially with media coverage, some leaders felt they needed to make sure that employees knew the importance of disclosure and were encouraged to report future errors: *“I think as far as what, clinically how we moved forward, I think a lot of, um, what I’ve done next in my team is t-to remind people to s-, ya know, speak up if you see something that doesn’t seem quite right.” -Facility employee.*

### Intervention characteristics: design of notification & packaging

Design and packaging refers to the presentation of the intervention and how accessible it is to users [14]. If the design and packaging is poor or perceived as poor, then it may be less likely to be used and may limit success. While the national disclosure policy outlines the types of events that must be communicated with patients and basic components of communication, it does not prescribe in detail how these communications must occur. Facilities work with national VA leadership to design a communications plan that the facility is responsible for executing. Some aspects of the plan receive more support from higher leadership, for example follow up with media and Congressional officials may be performed by local, regional or national public affairs staff. Many of the VA leaders and employees described a large volume of work that went into the planning for these patient communications without detailed guidance on best practices.

Patients provided feedback on the design of the intervention (the patient notification and testing clinics). The sites interviewed primarily notified patients using a certified, U.S. mail letter. Patients were invited to attend a special evaluation clinic for testing and a toll-free telephone hotline was available for questions to ensure both answers were accessible and uniform in message. Certified letters were used to communicate the adverse event, but the majority of patients interviewed were not satisfied with receiving a certified letter. Most preferred phone contact first, followed by a letter sent by regular mail. In addition, staff and patients asked for more information to be included in the letters/first contact. Patients wanted details about the event that could help them understand risk, and many frontline staff supported that: *“The contact was really confusing for patients. They weren’t given specifics and these gentlemen were people who could have understood specifics. One had no clue as to the extent of his risk.” -Facility employee.*

Patients and employees felt that those staffing the telephone hotline should be provided detailed information in order to be able to adequately answer questions. Many discussed the type/position of employee who should be making contact with patients. Preference was for employees with strong communications skills to make these first contacts, such as social workers or nurses.

Evaluation clinic design varied for each of the sites. All sites had dedicated space and staff for these special clinics. Some sites included an individual or group presentation first where hospital leadership presented patients with information about the event and showed the medical instruments that were used, if applicable. This gave patients the opportunity to ask questions before being tested. Some facilities also included opportunities for patients to meet with counselors, nurses, or chaplains one-on-one if they had additional questions or needed support. Several staff and leadership identified the need to have high-level leadership present in the testing clinics. These leaders made themselves available to meet with patients who were very angry or upset. Leadership presence was also important so that front-line staff felt supported. When leadership was absent, staff noted this as an issue: *“I didn’t see leadership in the clinic at all. I think (the Director) should have been down there and should have been making apologies to Veterans.” -Facility employee.*

### Intervention characteristics: execution

Tied in with the design of the notification, execution was also important. Timing was a key issue in execution. Many of the patients were not notified before the story was presented by the media, which was often thought to be due to a leak of the incident details rather than a planned press release. As a result, many patients saw news stories before they received notification letters. At some sites, there were multiple rounds of notifications due to patients inadvertently missed during initial reviews or expansion of the exposure cohort due to new information about the event or updated analyses. Internal communication was closely tied to execution. There were communications and approval delays at high levels of leadership that impacted the speed of the response: *“Facility staff knew what was happening from the media coverage but it took us a little longer to have internal communications ready, to get those approved by (central office). This is part of the central issue with communications.” -Facility leadership.* Coordination was essential for success in execution: *“When these things happen, you need consistent, confined, uniform, and targeted response.” -Regional leadership.*

The execution of the testing clinics was a positive aspect of notification. Staff were often able to arrange for temporary testing clinics quickly: *“We prepared this in only 5 days. We had to prepare reception in the lobby. Write scripts. Set up computers with privacy walls. Created a family support center. Completely moved primary care and turned it into a special care clinic.”* -Facility leadership. Facilities were able to test large numbers of patients, while providing optional counseling services. The speed of receiving testing results, however, was noted as an area that could be improved. Patients expressed that a 2-week waiting period for testing results led to increased anxiety.

A larger theme in timing of notifications emerged here. There were many stakeholder groups: patients, employees, advocacy groups, media, elected officials. Hospitals struggled with executing these notifications in a timely way. Employees, advocacy groups and elected officials must be notified in order to support the patients. However, notifying these groups before contacting patients can sometimes lead to media leaks. Hospitals all struggled with the timing of notifications but all recognized the importance of moving quickly to provide information to all of these stakeholders, and especially to patients. *“I would have involved stakeholders earlier. Give them a heads up. You might not have all the answers but you can say to them, this is what we know so far and we’ll follow through. Give them lots of updates.” -Facility leadership.*

### Process: engaging external stakeholders

Interview participants discussed elected officials, patient advocacy groups called Veteran Service Organizations (VSOs), and the media as external stakeholders. Stakeholders were engaged differently in the events studied which led to many lessons learned. VSOs are advocacy groups with extensive contact with patients and their families. VSO leadership were often quoted in media reports and VSOs frequently released their own statements on websites and social media. Some facilities alerted VSOs ahead of public releases about the event. Members of leadership at one facility gave in-person and telephone presentations to VSOs which were felt to be particularly helpful. These meetings gave leaders a chance to show VSOs the medical equipment that was involved in the event, demonstrate how the error occurred, and spend extensive time answering questions. VSOs can communicate information about testing and follow up to patients. Facilities that spent more time informing these stakeholders felt they benefited from the process: *“Before the press conference, we scheduled meetings with VSOs and congressional offices. We had a conference call for those who couldn’t join the in person meeting. I gave a full briefing and gave them firsthand information. We gave the bullet points on a handout. It was very helpful, more open. We had the right people at the table to answer their questions. There was no time limit for the meeting; I wanted to be sure they had all the info they needed.” –Facility leadership.*

Many interviewed talked about the importance of relationships with the media. Leaders and employees often reflected that relationships should be developed before an event occurs. This existing relationship can be beneficial in trying to make sure key information about the organization’s response is covered by reporters: *“The first lesson, it’s one I’ve believed in, every facility should develop strong relationships with the media well before the stuff hits the fan. Provide opportunity for the media to be invited to the medical center, offer them stories, these types of actions would preclude the media jumping on a “gotcha” type story. If they knew you, they wouldn’t put out these types of stories.” –Regional leader* Interviewees noted that the existing relationships can be hurt, however, when the facility does not respond quickly to media questions during an event.

Elected officials are another group that looks for detailed and timely information from VA regarding these events. Many facilities discussed delays in informing both local and national elected officials, which they perceived resulted in negative attention from these stakeholders, including negative comments in media stories. Many asked for elected officials to be informed early and continuously updated as the disclosure process unfolded. The delays in receiving approval for communication were often at higher levels of the VA, which involved long reviews of communication processes and a lack of consensus about how early to notify elected officials. Leaders struggled between notifying them early when information about the event was incomplete, or notifying them later with more complete information but much closer to the public notification period.

### Process: reflecting & evaluating

Reflecting and evaluating involves opportunities to provide feedback about the implementation and allows for debriefing [14]. Leaders and employees discussed their desire to share their experiences with others in the healthcare organization. Many talked about the disclosure policy and the start of the communication planning process. At that time, they looked for feedback from facilities that had conducted large-scale disclosures in the past. They reached out to staff at these facilities and asked them to share their best practices and lessons learned. *“Ask… who else has been through this and contact those sites. It is a huge undertaking and you don’t have to reinvent the wheel.”* -Facility employee. After the event, they wanted to formalize their own lessons learned for leadership and for facilities that would go through the process in the future. Many asked for a formal post process, expressing a desire to share their experience and tools they had developed with other facilities. *“Most facilities haven’t done anything like this before and they need the support.” -National leadership.*

### Intervention characteristics: costs

Costs of the intervention and implementation can take different forms, and exclude money, time, etc. in the CFIR analysis [14]. Overall, there were gains and losses following a large-scale adverse event. Many of the costs greatly impacted the facility and the VA as a whole, including significant impact on employee morale, damage to reputation, and loss of patient trust. Despite the costs, some interviewees were able to discuss the positives, including team building and improvements to the facility.

Some employees feared job loss and discipline during events: *“I saw a news report that the director lost his job. Monday morning, it was like a 100 % change. People throughout felt they could be out at any time.”* -Faclity employee. Leaders in particular felt they may be removed from their positions as a result of the event. In three facilities, leadership did change during and after event disclosure. The removal of leaders impacted the perception of job insecurity for other employees as well. It is important to note, however, that in cases where one provider was knowingly using unsafe practices, discipline and job loss was supported by interviewees.

Leaders, employees, and patients all discussed one of the greatest costs of disclosure of these events: trust in the healthcare organization. Trust was impacted negatively by delays in discovering issues and then in notifying patients. Further delays in obtaining testing results, use of letters instead of one-to-one contact, extensive negative media coverage, lack of detail about what happened and how organization is following up on issues further impacted trust and reputation.

Some patients interviewed, however, felt the disclosures of these adverse events increased trust in the organization: *“It actually made me feel better. They caught the mistake and improved. Everything is improved now. The clinic is cleaner. Everything is in top condition. I applaud them.” –Patient.*

These patients were pleased that the VA let them know about the event, even if it caused them significant distress. Many patients talked about learning more about the event and how the detail helped them to feel more confident that they had not been harmed and increased their trust. Some patients had negative past experiences with the organization and already had a low sense of trust. In those cases, the event served to confirm their lack of trust. Others who had more positive past experiences and higher trust in the organization were more trusting of efforts to disclose.

## Discussion

The Institute of Medicine’s *To Err is Human* called for dialogue and reporting of adverse events and efforts toward prevention of errors and improved safety culture [18]. This report stresses the difficulty but necessity of developing comprehensive approaches to patient safety. Individual disciplines, anesthesiology for example, have worked on identifying events and communicating with patients [19]. The call for open communication, both within the profession or the healthcare organization and with patients, about adverse events has long been support by the literature and individual disciplines [18, 19]. This literature often discusses the difficulty but importance of open communication following an adverse event. Although several organizations have released guidelines or policies for disclosure [20–22], the full impact on actual disclosure is unclear [23]. Much of this work has not differentiated between clinical adverse events and large-scale adverse events. Our work evaluating the implementation of a health care organization’s disclosure policy reveals many important gaps and successes. In addition, our focus on large-scale events identifies complex issues related to communicating with many patients in a short timeframe. How organizations respond to large-scale adverse events represents a type of stress test for the organizational culture. In a large healthcare organization like the VA, one large-scale adverse event affects not just one hospital but the whole system.

We found that existing networks and communications patterns become critical during large-scale disclosure when many groups need to be given key information quickly, including leaders and employees who will be implementing the policy and communicating with patients. If the existing communication channels are strong, the facility will be better equipped to carry out disclosures in a timely way. If there are communications issues prior to disclosure, this can negatively impact the outcomes of the implementation process. Strong communication networks are particularly important in a national system where an issue at one hospital can potentially affect the entire system.

The lasting impact of disclosure of large-scale adverse events can be damaging. We found that interviewees saw the costs of disclosure as loss of trust, and employees worried about job security. The VA disclosure policy was designed to promote transparency with patients regarding their healthcare; however, implementation issues can hinder this goal. This loss of trust may occur if patients do not receive a personal first contact and stakeholders are not informed in a timely way. When a facility delays responses or provides no response to media inquiries, this affects the perceived transparency of the organization. Providing timely responses with as much detail as possible may help maintain stakeholder relationships and improve quality of reporting. Many Veterans felt it was important that they were informed about the event, and they talked about how notification improved their trust in the VA. However, timing can be a major issue in a large organization with many layers. Leaders must set the tone, prioritize the response, and minimize the layers of approval in order to allow things to progress quickly.

The current VA large-scale disclosure policy is focused on ethics, not implementation. In support of existing research, we found that the design of the disclosure communication is critically important [1, 4]. Patients desired timely, personalized communication. However the process for doing this had not been outlined in the policy in detail sufficient to allow rapid implementation, and led to much planning work on behalf of the facilities. Patients stated that they preferred a disclosure process involving a personal phone contact from someone prepared to answer their questions. Letters, especially certified letters, were not viewed as a positive first contact for this patient group. Patients indicated that the speed and accuracy with which disclosure was implemented was also very important. Delays in the process were of great concern to them, and to other stakeholders, such as family members, and congressional staff.

Many facilities discussed the importance of existing organizational culture impacting the disclosure process. We found that a focus on the patient first in the existing culture helped this process. However, some leaders were concerned that their organization’s negative experiences with disclosure, as perceived by its employees and patients, may also lead to an organizational culture less inclined to effectively implement the disclosure policy. If the organization’s leadership does not emphasize the importance of disclosure and safety after a difficult disclosure process, the result could be hesitancy for employees to report similar large-scale adverse events affecting patients to their organization’s leadership in the future, thus leading to less transparency and follow-up of potential consequences to patients from the adverse event itself. Evaluating culture prior to any LSAEs may potentially be important in identifying problem areas and assessing organizational climate and safety. In future research, we plan to study employee survey data throughout the healthcare system to determine if there is a relationship between organizational climate and safety and the potential for a large-scale adverse event. Our goal is to identify large-scale adverse events prior to occurrence, using organizational climate and safety reports as a potential indicator of these events. Application of these indicators may lead to a focus on improving healthcare organization climate and safety and thus prevention of large-scale adverse events.

We experienced some limitations in studying the implementation of this large-scale disclosure policy. The VA has many levels of organizational structure involved in the implementation of this national policy which might not be the case in all healthcare organizations. It may be possible for smaller organizations to move more rapidly in approving patient disclosures and responding to stakeholder inquiries. The VA may also have some benefits in this process due to size. The organization is well equipped to test many patients for blood borne pathogen infections resulting from the large-scale adverse event in a short amount of time, when needed to assess for disease risk following an event. Additionally, we were only able to study nine past large-scale adverse events within this integrated healthcare system. To further our work in this area, we plan to develop a toolkit and a technical assistance program to assist healthcare facilities with implementing large-scale disclosures across the healthcare system, and study the impact of this toolkit on future implementation of this policy.

## Conclusions

Disclosure is an essential part of quality healthcare [2]. This study contributes to the literature on disclosures of large-scale adverse events by providing guidance on the additional elements of disclosure implementation needed to assist organizations in carrying out timely and coordinated disclosures to patients. The lessons learned from the experiences of the facilities in our study can help healthcare organizations of varying sizes implement their disclosure policies and provide timely communications to patients and other stakeholders in the future. All healthcare organizations may benefit from our finding about the importance of evaluating culture and communications prior to an adverse event and making improvements so that the disclosure implementation process will move smoothly when needed. Understanding patient preferences for communication (i.e. phone call first) can help any organization design effective disclosures. Employees and patients in this study were eager to share their experiences and insights for the design of disclosures to patients in the future. The VA has already acted on some of these lessons and continues to seek out improvements to the process [24]. Our prior work has shown that the design of disclosure communication may impact the ways in which the media represent the events [25]. Adverse events may also affect healthcare utilization [26]. Subsequently, attending to the ways in which the disclosure policy is carried out is critical. Our analysis reveals key constructs that one should attend to in order to optimize the disclosure communication.

## Additional file

Additional file 1: Interview Guides. Five interview guides used for the study. (DOCX 20 kb)

## Abbreviations

CFIR: Consolidated Framework for Implementation Research
VA: Department of Veterans Affairs, Veterans Health Administration

## References

[1] Prouty CD, Foglia MB, Gallagher TH (2013). Patients’ experiences with disclosure of a large-scale adverse event. J Clin Ethics.

[2] Dudzinski DM, Hebert PC, Foglia MB, Gallagher TH (2010). The disclosure dilemma—large scale adverse events. N Engl J Med.

[3] Holodniy M, Oda G, Schirmer P, Lucero CA, Khudyakov YE, Xia G (2012). Results from a large-scale epidemiologic look-back investigation of improperly reprocessed endoscopy equipment. Infect Control Hosp Epidemiol.

[4] Patel PR, Srinivasan A, Perz JF (2008). Developing a broader approach to management of infection control breaches in health care settings. Am J Infect Control.

[5] Fischhoff, B., Brewer, N., & Downs, J.S. (eds.). (2011). Communicating risks and benefits: An evidence-based user’s guide. Washington, DC: Food and Drug Administration.

[6] Kathleen M. Mazor, EdD; Steven R. Simon, MD; Robert A. Yood, MD; Brian C. Martinson, PhD; Margaret J. Gunter, PhD; George W. Reed, PhD; and Jerry H. Gurwitz, MD. Health Plan Members’ Views on forgiving medical errors. Am J Manag Care. 2005 Jan;11(1):49–52. 15697100

[7] Gallagher TH, Studdert D, Levinson W (2007). Disclosing harmful medical errors to patients. N Engl J Med.

[8] Veterans Health Administration Handbook 1004.08 Disclosure of Adverse Events to Patients [http://www.ethics.va.gov/Handbook1004-08.pdf]. Accessed 24 Oct 2016.

[9] Guh AY, Thompson ND, Schaefer MK, Patel PR, Perz JF (2012). Patient notification for bloodborne pathogen testing due to unsafe injection practices in the US health care settings, 2001–2011. Med Care.

[10] Elwy AR, Itani KM, Bokhour BG et al. Surgeons’ disclosures of clinical adverse events. JAMA Surg. 2016 Jul 20. doi: 10.1001/jamasurg.2016.1787. [Epub ahead of print] 10.1001/jamasurg.2016.1787 27438083

[11] Chafe R, Levinson W, Sullivan T (2009). Disclosing errors that affect multiple patients. CMAJ.

[12] Damschroder LJ, Lowery JC (2013). Evaluation of a large-scale weight management program using the consolidated framework for implementation research (CFIR). Implement Sci.

[13] Powell BJ, Proctor EK, Glisson CA, Kohl PL, Raghavan R, Brownson RC, Stoner BP, Carpenter CR, Palinkas LA.A mixed methods multiple case study of implementation as usual in children’s social service organizations: study protocol. Implement Sci. 2013 Aug 20;8:92. doi: 10.1186/1748-5908-8-92. 10.1186/1748-5908-8-92 PMC3751866 23961701

[14] Damachroder L, Aron D, Keith R, Kirsh S, Alexander J, Lowery J (2009). Fostering implementation of health services research findings into practice: a consolidated framework for advancing implementation science. Implement Sci.

[15] Consolidated Framework for Implementation Research. (2014, October 29). Retrieved September 14, 2016, from http://www.cfirguide.org. Accessed 24 Oct 2016.

[16] O’Brien BC, Harris IB, Beckman TJ (2014). Standards for reporting qualitative research: a synthesis of recommendations. Acad Med.

[17] Miles MB, Huberman M. Qualitative Data Analysis: A Sourcebook of New Methods. 2. Beverly Hills, CA: Sage Publications; 1994.

[18] Institute of Medicine. Committee on Quality of Health Care in America. To err is human: Building a safer health system. Washington, DC: National Academy Press, 2000.

[19] Anesthesia Patient Safety Foundation. Special issue: Dealing with adverse events. APSF Newsletter 2006;2. Accessed October 5 2016: http://www.apsf.org/newsletters/html/2006/spring/01adverse_event.htm

[20] McDonald T (2009). Error disclosure: Within a principled approach to adverse events. Am Soc Anesthesiol News lett.

[21] The Full Disclosure Working Group. When things go wrong: Responding to adverse events. A consensus statement of the Harvard Hospitals. Boston, MA: Massachusetts Coalition for the Prevention of Medical Errors, 2006. 10.1097/00129234-200607000-00001 16926690

[22] Conway J, Federico F, Stewart K, Campbell M. Respectful management of serious clinical adverse events. IHI Innovation series white paper. Cambridge, MA: Institute for Healthcare Improvement; 2010.

[23] Souter KJ, Gallagher TH (2012). The disclosure of unanticipated outcomes of care and medical errors: what does this mean for anesthesiologists?. Anesth Analg.

[24] Elwy AR, Bokhour BG, Maguire EM, Wagner TH, Asch SM, Gifford AL (2014). Improving healthcare systems’ disclosures of large-scale adverse events: a Department of Veterans Affairs leadership, policymaker, research and stakeholder partnership. J Gen Intern Med.

[25] Maguire EM, Bokhour BG, Asch SM (2016). Disclosing large scale adverse events in the US Veterans Health Administration: lessons from media responses. Public Health.

[26] Wagner TH, Taylor T, Cowgill E (2015). Intended and unintended effects of large-scale adverse event disclosure: a controlled before-after analysis of five large-scale notifications. BMJ Qual Saf.

