# Supplementary material for: Evaluating the implementation of a national disclosure policy for large-scale adverse events in an integrated health care system: identification of gaps and successes
Source: BMC Health Serv Res. 2016 Nov 11;16:648. doi: 10.1186/s12913-016-1903-7 (PMC5106838; doi:10.1186/s12913-016-1903-7)
Supplement: Additional file 1: — Interview Guides. Five interview guides used for the study. (DOCX 20 kb) [file 12913_2016_1903_MOESM1_ESM.docx]

Supplementary Files: **Evaluating the implementation of a national disclosure policy for large-scale adverse events in an integrated health care system: Identification of gaps and successes**

**Interview Guides**

**Leader Interview Guide**

*For Leadership that was in place at the time of the event:*

*These questions will be part of the half-hour semi-structured telephone interview that takes place prior to the Bedford VA research team conducting a visit to the site to conduct interviews with staff and Veterans.*

*“Thank you for agreeing to participate in this interview. We are interviewing you because of your expertise in this area, having gone through this large scale adverse event disclosure in recent years. Your expertise will help us, the research team, identify important and effective strategies for future medical center and VISN leaders who will need to disclose this kind of information to Veterans and their families in the future”.*

1. How did you find out about the adverse event?
   1. What were the local procedures for reporting?
   2. Have processes changed since the event
2. Can you describe what happened once the event was reported to you?
   1. What went well
   2. What was problematic
3. What went well with the disclosure process? Were there any positive results or outcomes that occurred due to this event and disclosure?
4. What were the greatest challenges you faced?
5. Who was involved in communicating with the media?
   1. How did that go?
6. Who else outside of the VA did you need to communicate with about the event?
   1. How did that go?
7. How did this affect the medical center?
   1. Which staff was affected by the event and the disclosure (this information will further inform which staff we seek to interview).
   2. What support was provided to staff?
8. How did this affect you personally?
   1. Did you deal directly with any patients or family members? What was that like?
9. What advice do you have for other VA leaders and administrators as a result of your experience?
10. What would you have done differently when communicating with Veterans and the public if you had to repeat this event again?
11. What additional support from the VISN (for medical center directors) or VA Central Office (for VISN directors) would have been most helpful to you regarding this event?

*For Leadership members who were not part of the event:*

1. What do you know about the adverse event notification?
   1. Were you part of the VA at the time?
   2. How did you learn about the event?
2. What is your understanding of the facility response to the event?
   1. What went well
   2. What was problematic
3. Have you been involved in any follow up from the event?
4. How did this affect the medical center’s reputation?
5. What would you have done differently when communicating with Veterans and the public if you had to repeat this event again?
6. What concerns do you have about future event notifications?
7. What support from the VISN (for medical center directors) or VA Central Office (for VISN directors) would be most helpful for this type of event?

**Employee Interview Guide**

*These questions are meant* ***as a guide.***

***Please begin by stating:*** *“In large health care systems like the VA, sometimes things happen that effect patients. We are interested in finding out more about what [the event] was like for staff. There are no right or wrong answers; we are just trying to get your opinion on the events. Everything you say will be kept confidential, and your participation is completely voluntary. If you want to stop this interview at any time, we can do that, or we can skip any questions that you don’t want to answer.”*

1. Can you tell me how you first found out about [adverse event]?
2. What did you think when you first heard about this?
   1. What kind of reactions did you have? (follow up question if needed)
   2. What kind of feelings do you have? (follow up question if needed)
   3. What was the mood here in your clinic (or facility)?
3. What kind of communication did you receive from the hospital or VISN leadership regarding this event?
   1. Was there an apology from leadership? If so, what did they say?
   2. How did the front line providers find out? Were you involved in that communication?
4. What has this been like for staff and the clinic?
   1. Staff relationships?
   2. Relationships with patients?
   3. Job security?
5. What was this like for you personally?
   1. Were you worried about your job at the time?
6. Did patients talk to you about [this event]?
   1. What kinds of questions did they ask?
   2. What was that like for you?
      1. How prepared did you feel to respond to the questions asked?
   3. How did the patients find out? What did you think about the communication to patients?
7. What kind of communication did you have with the media about this event?
8. What kind of support did you receive from VA leadership that you found important?
9. What kind of support did you feel you needed but did not receive?
10. Is there anything else you would like to tell me about this event and the disclosure of this event?

**Patient Interview Guide**

*These questions are meant* ***as a guide.***

***Please begin by stating:*** *“We want to understand what it’s like for patients when they’re told that something has gone wrong at their hospital. So we want to talk to you today about (the event) and how it was for you and your family. There are no right or wrong answers; we are just trying to get your opinion on the events. Everything you say will be kept confidential, and your participation is completely voluntary. If you want to stop this interview at any time, we can do that, or we can skip any questions that you don’t want to answer.”*

1. Today, we are talking about when there was an issue with [event] at the X VA. Can you tell me how you found out about that?

***Prompt:***

- What was your first reaction?
- What did you think?
- How did you feel when you found out?
- If this were to happen again, how would you like to find out?

1. What did you do when you found out?

***Prompt:***

- What did the VA do afterwards? *(try to find out if Dr. initiated blood test)*
- Did you see your Dr? *(try to find out if patient scheduled an appointment after finding out)*
- What happened after that?
- Did you speak with anyone else about the event? What did you speak about?
- Is there anything else that you think should have been done?

1. What did people at the VA say to you about the event?

- Do you feel you had an opportunity to share your thoughts with the VA?

1. What other ways did you hear about the event?

***Prompt:***

- Did reading/seeing/hearing this change the way you saw the event? How?

1. How important do you think it was that you were told about this event?
   - Would you feel the same way if this event did not put you at risk for HIV or hepatitis? Ex. Bacterial infection that would have already passed by the time you found out about the event.
2. How did you feel about the VA at that time?

***Prompt:***

- How did you feel about the VA as a whole and how did you feel about the facility?
- What/who made you feel that way?
- Has something happened since then to change how you feel?

1. How do you feel about the VA now?

***Prompt:***

- How do you feel about the VA as a whole and how do you feel about the facility?
- Do you still use the VA for the same services?
- Do you still see your Dr?
- Do you have any concerns about using the VA? What are they?

*At your discretion, show patient the notification letter.*

1. Do you remember receiving this?
2. What was the most important part of the letter?
3. Anything in the letter you thought shouldn’t have been said?
4. Was there anything you think was missing from the letter?

*Repeat any questions the patient had difficulty answering before seeing the letter.*

1. Sometimes these events happen and it’s a long time before notification occurs. What do you think about notifying family members if, in the meantime, the patient has passed away?
2. Is there anything else you would like to tell me?

**Friend/Family Member Interview Guide**

These questions are meant **as a guide.**

**Please begin by stating:** “We want to understand what it’s like for patients and families when they’re told that something has gone wrong at their hospital. So we want to talk to you today about (the event) and how it was for you and your family. There are no right or wrong answers; we are just trying to get your opinion on the events. Everything you say will be kept confidential, and your participation is completely voluntary. If you want to stop this interview at any time, we can do that, or we can skip any questions that you don’t want to answer.”

1. What do you remember about [the event]?

***Prompt:***

- What did your friend/family member tell you? Did you see the notification letter?
- What did you think?
- How did you feel when you found out?
- What was your first reaction?

1. What did you do when you found out?

***Prompt:***

- What advice to you give your friend/family member?
- Did you speak with anyone else about the event? What did you speak about?
- Is there anything else that you think should have been done?

1. What other ways did you hear about the event?

***Prompt:***

- Did reading/seeing/hearing this change the way you saw the event? How?

1. How important do you think it was that your family member was told about this event?
2. How did you feel about the VA at that time?

***Prompt:***

- VA as a whole and the facility
- What/who made you feel that way?
- Has something happened since then to change how you feel?

1. How do you feel about the VA now?

***Prompt:***

- VA as a whole and the facility
- Are you comfortable with your friend or family member using VA services?

1. Is there anything else you would like to tell me?

**At your discretion, show patient the notification letter.**

1. Do you remember seeing this?
2. What was the most important part of the letter?
3. Anything in the letter you thought shouldn’t have been said?
4. Was there anything you think was missing from the letter?
5. Sometimes these events happen and it’s a long time before notification occurs. What do you think about notifying family members if, in the meantime, the patient has passed away?

**Congressional Staffer Interview Guide**

*These questions are meant* ***as a guide.***

***Please begin by stating:*** *“In large health care systems like the VA, sometimes things happen that effect patients. We are interested in finding out more about what these events are like for Congressional staff. There are no right or wrong answers; we are just trying to get your opinion on the events. Everything you say will be kept confidential, and your participation is completely voluntary. If you want to stop this interview at any time, we can do that, or we can skip any questions that you don’t want to answer.”*

1. Can you tell me how you first find out about VA large scale adverse events (when something goes wrong with health care at a VA medical facility, such as the insulin pen event at Buffalo VA)?
2. What happens after you find out? What do you do?
3. What kind of communication did you receive from VHA and VA leadership regarding events?
   1. Is there something that you feel goes well when communicating with the VA about these events?
   2. Is there something that you think could improve the communication process during these events?
4. Do you have any suggestions for how the VA can communicate with congressional staffers before, during and after these events?
5. Is there anything else you would like to tell me about the communication of these events?
